# Molecular Detection of *Babesia* spp. (Apicomplexa: Piroplasma) in Free-Ranging Canids and Mustelids From Southern Italy

**DOI:** 10.3389/fvets.2019.00269

**Published:** 2019-08-23

**Authors:** Mario Santoro, Clementina Auriemma, Maria Gabriella Lucibelli, Giorgia Borriello, Nicola D'Alessio, Giovanni Sgroi, Vincenzo Veneziano, Giorgio Galiero, Giovanna Fusco

**Affiliations:** ^1^Istituto Zooprofilattico Sperimentale del Mezzogiorno, Portici, Italy; ^2^Department of Veterinary Medicine and Animal Productions, Università degli Studi di Napoli “Federico II”, Naples, Italy

**Keywords:** *Vulpes vulpes*, *Meles meles*, *Canis lupus*, *Babesia vulpes*, badger-associated *Babesia* spp., babesiosis, tick-borne disease

## Abstract

Babesiosis is an emerging tick-borne disease caused by apicomplexan parasites with widespread geographical distribution and various wildlife species as reservoir hosts. The aims of this study were to investigate the prevalence and assess the role of free-ranging canids and mustelids in the maintenance of *Babesia* spp. in southern Italy. PCR analysis of splenic samples targeting the 18S rRNA gene revealed the presence of *Babesia* spp. in 36 of 82 (43.9%) red foxes (*Vulpes vulpes*) including 29 (58%) from Campania region and seven (21.8%) from Calabria region, in seven of 13 (53.8%) Eurasian badgers (*Meles meles*), and in one of 13 (7.7%) gray wolves (*Canis lupus*). Samples from other host species including 9 Eurasian otters (*Lutra lutra*), 1 stone marten (*Martes foina*), 1 least weasel (*Mustela nivalis*), and 1 European polecat (*Mustela putorius*) tested *Babesia* spp. negative. Sequence analysis of the 18S rRNA gene demonstrated the presence of *B. vulpes* in the red fox and two sequence types of badger-associated *Babesia* spp. in the Eurasian badger. The *Babesia* sp. sequence detected in the gray wolf was identical to a badger-associated *Babesia* sp. This study shows that the number of *Babesia* spp. infecting free-ranging carnivores in Italy is higher than currently believed, and suggests that these hosts may play an important role in the maintenance of the sylvatic cycle of these parasites. It is the first report of badger-associated *Babesia* spp. in Italy and in a gray wolf.

## Introduction

Babesiosis is a tick-borne disease caused by haemoprotozoan organisms of the genus *Babesia* (Apicomplexa: Piroplasma), and several wildlife species may represent important reservoir hosts. According to the most recent studies, *Babesia* spp. of carnivore mammals are divided into three main groups including the *Babesia microti* group infecting felids, canids, mustelids and procyonids; the prototheilerid group infecting felids, canids, herpestids and hyaenids; and *Babesia* sensu stricto group infecting canids, procyonids, and ursids (1–3).

In free-ranging carnivores in Europe, infection with at least three *Babesia* spp. has been documented including *B. canis* in the gray wolf (*Canis lupus*) (4), *B. canis* and *B. vulpes* (this latter reported in the literature as *B*. “Spanish dog isolate,” *B. microti*-like, *B. annae*, and *Theileria annae*) (5) in the red fox (*Vulpes vulpes*), and *B. vogeli* in a stone marten (*Martes foina*). Moreover, at least two genotypes named badger-associated *Babesia* spp. in the Eurasian badger (*Meles meles*) (1, 3, 6) and a genotype that clustered together to *B. vulpes* isolates [FJ225390.1 (7)] have been reported. An additional *Babesia* genotype in the European wildcat (*Felis silvestris silvestris*) from Bosnia and Herzegovina (8) has been also reported. *B. vulpes* and badger-associated *Babesia* spp. are included in the *B. microti* group; *B. canis* and *B. vogeli* are included in the *Babesia* sensu stricto group (1–3).

There are few studies focusing on the *Babesia* spp. in free-ranging carnivores other than red fox in Europe, and none in Italy (1, 3, 6, 8). Due to recent reports of DNA sequences than could represent new species and the overall lack of prevalence surveys in most carnivore species and geographic locations, it is likely that the number of *Babesia* spp. infecting free-ranging carnivores is higher that currently believed (1, 6–10).

Among *Babesia* species in free-ranging carnivores in Italy, only three recently studies from northern and central Italy reported the presence of *B. vulpes* in the red fox (11–13). All those studies suggested that regional factors as climatic and landscape variables linked to tick vector abundance and wild carnivore population densities may exist (11–13). Here we perform a molecular survey to study the occurrence of *Babesia* spp. in free-ranging canids and mustelids in the southern Italy where emerging tick-borne pathogens (i.e., *B. canis, B. gibsoni, B. vogeli, Ehrlichia canis, Anaplasma* spp., *Borrelia burgdorferi* spp., and *Rickettia* spp.) have been recently reported in domestic and free-ranging carnivores (14–18).

## Materials and Methods

### Sample Collection

Carcasses of 120 free-ranging carnivores including members of two families (Canidae and Mustelidae) obtained from the southern Italy were used for this study (Table 1). Carcasses were all road-killed animals except for the red foxes obtained during the official hunting season from local hunters. Carcasses were all obtained between January 2017 and December 2018 except for six Eurasian otters (*Lutra lutra*) obtained between March 2004 and December 2016 [see also (16)].

**Table 1:** Carnivore species, region of sampling and positive individuals for *Babesia* spp. in southern Italy.

**Host species**	**Locality**	***n***	**Positive n**	**Prevalence of infection (%)**
Red fox (*Vulpes vulpes*)	Calabria	32	7	21.8
	Campania	50	29	58
Gray wolf (*Canis lupus*)	Campania	13	1	7.7
Eurasian badger (*Meles meles*)	Campania	13	7	53.8
Eurasian otter (*Lutra lutra*)	Basilicata	1	0	0
	Calabria	2	0	0
	Campania	6	0	0
Stone marten (*Martes foina*)	Campania	1	0	0
European polecat (*Mustela putorius*)	Campania	1	0	0
Least weasel (*Mustela nivalis*)	Campania	1	0	0

### Molecular Analyses

Spleen samples were collected at post-mortem examination and stored at −20°C for a week until DNA extraction. Genomic DNA was extracted from 25 mg of spleen with the QIAamp DNA mini KIT (QIAGEN) following manufacturer's instructions. All samples were screened for the presence of *Babesia* spp. by PCR targeting the 18S rRNA gene as described by Casati et al. (19). To assess the analytical sensitivity of the PCR assay, the DNA concentration was determined by biophotometry (BioPhotometer plus, Eppendorf, Italy). The extract was then serially diluted 10-fold in sterile water starting from 774 μg/ml, and each dilution was used as template for the PCR reaction. Briefly, reactions were performed in a total volume of 25 μL containing HotStar HiFidelity PCR Buffer 1X, 1 ul of HotStar HiFidelity DNA Polymerase, 0.6 μM of each primer, and 3.5 μL of DNA. The primers used were BJ1 (5′-GTC TTGTAATTGGAATGATGG-3′) and BN2 (5′-TAGTTTATGGTTAGGACTACG-3'). Amplification conditions included an initial denaturation/activation step at 95 °C for 5 min, followed by 35 cycles of denaturation at 94 °C for 1 min, annealing at 55 °C for 1 min and extension at 72°C per 2 min, and a final extension step at 72°C for 5 min. Amplicons (486–520 bp) were resolved by automated capillary electrophoresis with the instrument QIAxcel (QIAGEN). Amplicons were subsequently purified and sequenced in both directions using the same primers as for PCR, employing the Big Dye Terminator Cycle Sequencing Kit v1.1 (Thermo Fischer Scientific, USA) in the automated sequencer 3500 Genetic Analyzer (ThermoFisher). Sequences were aligned using BIOEDIT software and compared with those available in GenBank (BLAST—http://blast.ncbi.nlm.nih.gov/Blast.cgi). Phylogenetic analyses were performed with the Neighbor-Joining method, using MEGA 6.0 (20, 21).

## Results

Among DNA extracts, 36 of 82 (43.9%) samples of red fox, seven of 13 (53.8%) samples of Eurasian badger, and one of 13 (7.7%) samples of gray wolf were PCR-positive for *Babesia* spp. (Table 1). The amplicon detection limit for the PCR assay was 77.4 μg/ml corresponding to 1.9 μg/reaction.

Sequences from the red foxes (520 bp) were all identical and shared a similarity of 100% with *B. vulpes* 18S rRNA gene sequences found in GenBank (KY175167.1, KY175166.1). In the Eurasian badger and gray wolf we obtained six sequences belonging to two sequence types. The first type (515 bp) was found in four Eurasian badgers and a gray wolf (from MK742770 to MK742774) and showed a similarity of 100% with *Babesia* sp. badger type A 18S rRNA gene sequences found in China (MG799845.1, MH844530.1) and Spain (KT223484.1). The second one (486 bp) was found in a single Eurasian badger (MK742775), and showed a similarity of 100% with a *Babesia* sp. 18S rRNA gene sequence (JX679177.1) found in nymph individuals of the tick *Ixodes canisuga* collected from a red fox in Germany, and in an Eurasian badger in China (MG799847.1).

**Figure 1:**
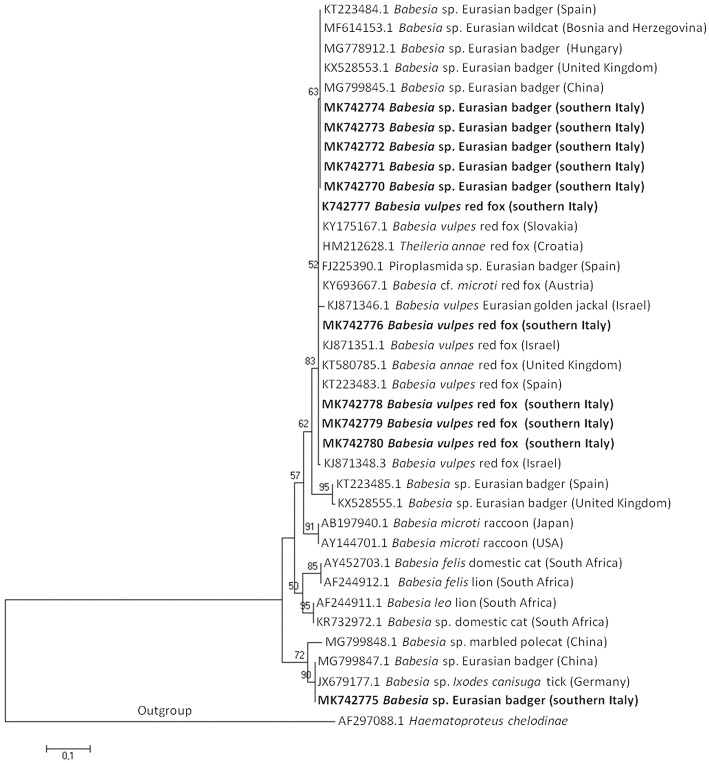
The evolutionary history of *Babesia microti* group DNA sequences was inferred using the Neighbor-Joining method. The optimal tree with the sum of branch length = 1,76412028 is shown. The percentage of replicate trees in which the associated taxa clustered together in the bootstrap test (1,000 replicates) is shown above the branches. The tree is drawn to scale, with branch lengths in the same units as those of the evolutionary distances used to infer the phylogenetic tree. The evolutionary distances are in the units of the number of base substitutions per site. Sequences are presented by GenBank accession number, *Babesia* species, host species, and geographical location in brackets. Isolate from this study are in bold.

A Phylogenetic tree of the 18S rRNA gene of *Babesia* spp. of *microti* group using the Neighbor-Joining method, showed that *B. vulpes* sequences from this study clustered together with *B. vulpes, B*. sp. Spanish dog isolate, *B. annae, T. annae*, and *B. microti*-like piroplasm confirming the synonymy with those species. The first type sequence detected in the Eurasian badger and gray wolf clustered together with *Babesia* sp. badger type A and *Babesia* sp. Meles Hu1 found in European countries; the second type sequence detected in a Eurasian badger clustered together with a *Babesia* sp. found in Germany and China (Figure 1).

## Discussion

In this study, the splenic samples of seven free-ranging carnivore species from the southern Italy were molecularly screened for the presence of *Babesia* spp. The DNA of three *Babesia* spp. all belonging to the *B. microti* group was detected.

Published data regarding *Babesia* spp. in free-ranging carnivores in Italy is limited to few studies that detected *B. vulpes* in the red fox only. In the red fox in Italy, *B. vulpes* prevalence was highly variable: 0.98% in north-western (11), 54% in north-eastern (13), and 22.88% in central Italy (12). PCR prevalence of *B. vulpes* in this survey differed depending on geographical locality. In Campania region, *B. vulpes* prevalence was almost three times higher than that reported in Calabria region (58 vs. 21.8%), but it was lower than prevalence found in Portugal [69.2% (22)] and Galicia, Spain [from 61 to 79.8% (23)].

Prevalence of badger-associate *Babesia* spp. in the Eurasian badger has been reported to range from 21.3 to 53.2% in Scotland (9), from 20 to 35.2% in Spain (6, 7), and it was 66.7% in Hungary (3). Prevalence of badger-associated *Babesia* spp. in the Eurasian badger in this study was among the higher prevalence values recorded to date.

Difference in *Babesia* spp. prevalence suggests regional differences in tick species diversity and abundance, and infection rate of the pathogen in ticks may influence the dynamic of *Babesia* spp. infection in mammalian hosts. In a recent study from Spain, Checa et al. (23) found significant differences in prevalence of *B. vulpes* infection among red fox populations of Galicia provinces suggesting that humid climates found in those provinces with highest prevalence rates are capable of sustaining higher burdens of ixodid ticks, which in turn may lead to the increased risk of tick-borne diseases. Differences in prevalence of *B. vulpes* between Campania and Calabria regions indicate a lower circulation of the parasite in Calabria region and could be related to the highest red fox density and tick vector abundance in those localities of the Campania region where the red foxes were obtained. A great abundance of potential hosts is an important factor for tick expansion and a potential for the spread of babesiosis (3, 4, 23). Southern Italy represents an endemic area for several tick-borne pathogens infecting domestic and free-ranging carnivores showing distribution corresponding to known distribution of its tick vectors (14–18). DNA of *B. vulpes* has been detected in many European countries in few tick species infecting the red fox including *I. canisuga, I. hexagonus, I. ricinus, Rhipicephalus sanguineus* s.l., and *Dermacentor reticulatus;* all these species are present in southern Italy except the last one. *I. hexagonus* is considered the most likely vector for *B. vulpes* (23, 24).

Despite the frequent contacts occurring between domestic dogs (as hunting dogs and sheepdogs) and red foxes, and the few ixodid tick vectors they share, infection of dogs and other free-ranging carnivores with *B. vulpes* occurred occasionally suggesting the red fox as its main natural host (1, 25). A recent survey of *Babesia* spp. in 1,311 hunting dogs in the Campania region of southern Italy revealed only the presence of *B. canis, B. gibsoni*, and *B. vogeli* (18). It suggests that at least in this region the red foxes and hunting dogs tend to acquire different *Babesia* spp.

In Europe, at least two DNA sequences of badger-associated *Babesia* spp. that could be undescribed *Babesia* species (named *Babesia* sp. isolate badger type A or *Babesia* sp. isolate Meles Hu1, and *Babesia* sp. isolate badger type B) have been reported in the Eurasian badger in Spain (6), Scotland (9), and Hungary (3) (see phylogenetic tree in Figure 1). An additional sequence type available in GenBank corresponds to a *Babesia* sequence detected in a Eurasian badger in Xinjiang, China (*Babesia* sp. badger isolate Badger-1; MG799847.1), and in an *I. canisuga* tick collected in a red fox in Germany (*Babesia* sp. 4 NAN2012; JX679177.1). Four out of five badger-associated *Babesia* spp. sequences detected in the present study in the Eurasian badger were identical to type A sequences found in China and Spain; the other one (MK742775) was identical to those found in China (MG799847.1) and Germany (JX679177.1). The high homology among sequences here found and those deposited in GenBank suggests that the parasites may circulate among Eurasian badgers across Europe.

Contrary to that observed for *B. vulpes*, a recent study demonstrated that the infection of badger-associated *Babesia* spp. may occur in dogs showing clinical signs of babesiosis (3). The usage of dogs during badger hunting in Hungary was considered as a plausible explication for the dog infections (3), since few *Babesia* spp. within the *microti* group may be transmitted orally by direct contact with infected blood (26, 27). The absence of the badger-associated *Babesia* spp. infection in hunting dogs from southern Italy may be linked to two main facts: (a) in Italy the badger hunting is an unperformed practice; (b) the hunters treated regularly their dogs with ectoparasiticides, as a result of being informed of the risks of pathogen transmission by ticks and other vectors (17, 18).

To our knowledge there are only two previous reports of *Babesia* infection in the gray wolf (4, 28). Erdelyi et al. (28) described a fatal infection by *B. canis* in two male captive gray wolves in a private farm in Hungary. Beck et al. (4) reported the infection by *B. canis* in seven of 108 gray wolves (including one captive and six free-ranging) in Croatia. The *Babesia* positive gray wolf found in the present study was a road killed adult female collected in January 2017 in Montemarano (Avellino municipality). It represents the first molecular confirmed case of a badger associated *Babesia* infection in a free-ranging gray wolf. It is plausible to suppose that the gray wolf was infected via blood-sucking ticks or alternatively preying on an infected Eurasian badger similarly to that hypothesized by Hornok et al. (3) for hunting dogs in Hungary.

In conclusion, the red fox and the Eurasian badger in southern Italy exhibit a high prevalence of infection caused by *B. vulpes* and badger-associated *Babesia* spp., respectively. It suggests that these hosts may play an important role in the maintenance of the sylvatic cycle of these parasites. This represents the first detection of badger-associated *Babesia* spp. in Italy. The detection of a badger-associated *Babesia* sp. in a gray wolf deserves further studies to understand the gray wolf role in the maintenance of this *Babesia* strain in the wild.

## Data Availability

Representative nucleotide sequences of *Babesia* spp. found in this study have been submitted to GenBank and are available with the following accession numbers: from MK742776 to MK742780 for B. vulpes; MK742775 for the Babesia sequence detected from a single Eurasian badger; from MK742770 to MK742774 for the Babesia sequences detected in four Eurasian badger and a gray wolf.

## Ethics Statement

Ethical review and approval was not required for the animal study because The IZSM is accredited by the Italian Ministry of Health to perform systematic surveys on infectious diseases of domestic and free-ranging animals. Procedures for this study were performed in accordance with the guide for the care and use of animals by the Italian Ministry of Health.

## Author Contributions

MS, GG, and GF: experimental conception and design. GS, ND, and VV: collection of samples. CA, ML, and GB: molecular analysis. MS: paper writing. All authors read and approved the final manuscript.

### Conflict of Interest Statement

The authors declare that the research was conducted in the absence of any commercial or financial relationships that could be construed as a potential conflict of interest.
